# The Impact of COVID-19 on Older Adults

**DOI:** 10.1093/geroni/igab046.2743

**Published:** 2021-12-17

**Authors:** Rachel Ungar, Rifky Tkatch, Jane Huang, Sandra Kraemer, James Schaeffer, Charlotte Yeh

**Affiliations:** 1 UnitedHealth Group, Minnetonka, Minnesota, United States; 2 UnitedHealth Group, Oak Park, Michigan, United States; 3 UnitedHealthcare, Minnetonka, Minnesota, United States; 4 AARP Services, Inc., Washington, District of Columbia, United States

## Abstract

Background: The onset of the COVID-19 pandemic has dramatically influenced the health and well-being of older adults. Changes in lifestyle patterns has required reframing communication habits and learning new skills to maintain social connections and access healthcare. Objectives: To assess 1) well-being measured prior to and during the COVID-19 era; and 2) use and comfort level of technology for social interactions and telehealth visits during this time. Methods: A mailed survey to a randomly selected national sample (>65) during the summers of 2018, 2019, and 2020. Measures included mental and physical well-being and various psychosocial measures. For 2020, questions related to COVID-19 and the use of technology were included. Results: A total of 4,696 (2018), 3,976 (2019) and 2,726 (2020) responded to these surveys (response rate ~27%). Overall, most constructs remained stable despite the ongoing pandemic. Most respondents reported average or high resilience (90%), high purpose (48%), stable social networks (76%), and low stress (55%). However, loneliness increased during 2020 (57%). Respondents who used technology were more likely to connect with family and friends. Only 43% reported high comfort with using technology, with older age (>75) less comfortable. At the time of the survey 37% had not seen a healthcare provider through telehealth services, and 15% felt their healthcare needs were not met by a telehealth experience. Conclusion: Results demonstrate that respondents were doing well during COVID-19. Yet increases in loneliness and greater technology needs to stay socially connected and to access healthcare may result in negative long-term health outcomes.

